# Supplementary material for: RNA promotes phase separation of glycolysis enzymes into yeast G bodies in hypoxia
Source: eLife. 2020 Apr 16;9:e48480. doi: 10.7554/eLife.48480 (PMC7162659; doi:10.7554/eLife.48480)
Supplement: Transparent reporting form [file elife-48480-transrepform.docx]

***eLife’s* transparent reporting form**

We encourage authors to provide detailed information *within their submission* to facilitate the interpretation and replication of experiments. Authors can upload supporting documentation to indicate the use of appropriate reporting guidelines for health-related research (see [EQUATOR Network](http://www.equator-network.org/%20)), life science research (see the [BioSharing Information Resource](https://biosharing.org/)), or the [ARRIVE guidelines](http://www.plosbiology.org/article/info:doi/10.1371/journal.pbio.1000412) for reporting work involving animal research. Where applicable, authors should refer to any relevant reporting standards documents in this form.

If you have any questions, please consult our Journal Policies and/or contact us: [editorial@elifesciences.org](mailto:editorial@elifesciences.org).

**Sample-size estimation**

- You should state whether an appropriate sample size was computed when the study was being designed
- You should state the statistical method of sample size computation and any required assumptions
- If no explicit power analysis was used, you should describe how you decided what sample (replicate) size (number) to use

Please outline where this information can be found within the submission (e.g., sections or figure legends), or explain why this information doesn’t apply to your submission:

Mass spectrometry was performed in duplicate.

Between two and four biological replicates were performed for imaging experiments.

For quantification of G body formation, at least 100 cells were analyzed per condition per replicate. Full sample sizes are included for each replicate in the source data file.

Sample sizes are described in figure legends.

**Replicates**

- You should report how often each experiment was performed
- You should include a definition of biological versus technical replication
- The data obtained should be provided and sufficient information should be provided to indicate the number of independent biological and/or technical replicates
- If you encountered any outliers, you should describe how these were handled
- Criteria for exclusion/inclusion of data should be clearly stated
- High-throughput sequence data should be uploaded before submission, with a private link for reviewers provided (these are available from both GEO and ArrayExpress)

Please outline where this information can be found within the submission (e.g., sections or figure legends), or explain why this information doesn’t apply to your submission:

All imaging experiments and G body RIPs have two to four biological replicates. G body RIP sequencing has two biological replicates. Biological replicates are defined as experiments performed independently arising from separate starter cultures and cultured in independent hypoxic environments.

Data were not excluded from analysis in quantification of focus formation. For fitting of Gaussian distributions, if non-linear least squares fitting failed to produce a solution, data points were omitted from analysis. Frequently, this was due to cells that had moved while a Z-stack was being captured, thus producing elongated punctae in maximum intensity projections that were inaccurate. For FRAP analysis, we only considered cells where at least 60% of the G body was bleached.

For sequencing and mass spectrometry analysis, detailed descriptions of data analysis appear in materials and methods. High throughput sequencing analysis is described in the subsections: “Yeast PAR-CLIP-seq (Pfk2, Eno1, Fba1)” and “Pfk2, Fba1, and Eno1 binding site generation”. Additional information is included in the figure legends. For mass spectrometry, Figure Legend 1 includes a detailed description of parameters used for candidate description.

Sequencing data associated with Figure 2 and Figure 1-Figure Supplement 1 have been uploaded to GEO (accession GSE65992). Sequencing data associated with Figure 3 and Figure S2 have been uploaded to GEO (accession GSE145881).

**Statistical reporting**

- Statistical analysis methods should be described and justified
- Raw data should be presented in figures whenever informative to do so (typically when N per group is less than 10)
- For each experiment, you should identify the statistical tests used, exact values of N, definitions of center, methods of multiple test correction, and dispersion and precision measures (e.g., mean, median, SD, SEM, confidence intervals; and, for the major substantive results, a measure of effect size (e.g., Pearson's r, Cohen's d)
- Report exact p-values wherever possible alongside the summary statistics and 95% confidence intervals. These should be reported for all key questions and not only when the p-value is less than 0.05.

Please outline where this information can be found within the submission (e.g., sections or figure legends), or explain why this information doesn’t apply to your submission:

All statistical analyses are described in the figure legend of each figure. Raw p-values are available in source data files.

(For large datasets, or papers with a very large number of statistical tests, you may upload a single table file with tests, Ns, etc., with reference to sections in the manuscript.)

**Group allocation**

- Indicate how samples were allocated into experimental groups (in the case of clinical studies, please specify allocation to treatment method); if randomization was used, please also state if restricted randomization was applied
- Indicate if masking was used during group allocation, data collection and/or data analysis

Please outline where this information can be found within the submission (e.g., sections or figure legends), or explain why this information doesn’t apply to your submission:

Groups were allocated according to different genotypes or experimental treatments (for example, vector controls in Figure 4A were a different group from cells with a plasmid expressing Pfk2-MqsR-Flag, and within these, separate groups existed for each concentration of copper sulfate used). This information is clear from the figures.

Samples were not masked during collection or analysis.

**Additional data files (“source data”)**

- We encourage you to upload relevant additional data files, such as numerical data that are represented as a graph in a figure, or as a summary table
- Where provided, these should be in the most useful format, and they can be uploaded as “Source data” files linked to a main figure or table
- Include model definition files including the full list of parameters used
- Include code used for data analysis (e.g., R, MatLab)
- Avoid stating that data files are “available upon request”

Please indicate the figures or tables for which source data files have been provided:

For Figure 1, Supplementary File S1 includes all source data.

For Figure 2, 3 and Figure 1 Figure Supplement 1, our GEO submission includes all source data.

Source data files are included with source data of all image quantification.
